# 4-[(*E*)-(4-Fluoro­benzyl­idene)amino]­benzoic acid

**DOI:** 10.1107/S1600536811052275

**Published:** 2011-12-21

**Authors:** Blanca M. Muñoz-Flores, Víctor M. Jiménez Pérez, Rosa L. Santillan, Maria Eugenia Ochoa, Noemi Waksman

**Affiliations:** a Facultad de Ciencias Químicas, Universidad Autónoma de Nuevo León, San Nicolás de los Garza, 66451 Nuevo León, Mexico; b Departamento de Química, Centro de Investigación y de Estudios Avanzados, del Instituto Politécnico Nacional, Apartado Postal 14-740, 07000 México, DF, Mexico; c Departamento de Química Analítica Facultad de Medicina, Universidad Autónoma de Nuevo León, León PO Box 2316, 64841 Nuevo León, Mexico

## Abstract

In the title compound, C_14_H_10_FNO_2_, the benzene rings make a dihedral angle of 57.50 (13)°, and the molecule has an *E* configuration about the C=N bond.  In the crystal, molecules are linked *via* pairs of O—H⋯O hydrogen bonds, forming inversion dimers.

## Related literature

For the synthesis, properties and uses of 4-(benzyl­idene­amino)­benzoic acid, see: Borisova *et al.* (2007 ▶); Schiff (1864 ▶); Innocenzi & Lebeau (2005 ▶); Muñoz-Flores *et al.* (2008 ▶).
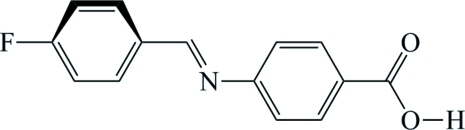


## Experimental

### 

#### Crystal data

C_14_H_10_FNO_2_
*M* *_r_* = 243.24
Monoclinic, 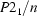

*a* = 12.2787 (5) Å
*b* = 5.6264 (2) Å
*c* = 17.2874 (8) Å
β = 105.833 (2)°
*V* = 1148.99 (8) Å^3^
*Z* = 4
Mo *K*α radiation
μ = 0.11 mm^−1^
*T* = 293 K
0.23 × 0.2 × 0.15 mm

#### Data collection

Nonius KappaCCD diffractometer
9975 measured reflections
2002 independent reflections
1226 reflections with *I* > 2σ(*I*)
*R*_int_ = 0.186

#### Refinement

*R*[*F* ^2^ > 2σ(*F* ^2^)] = 0.062
*wR*(*F* ^2^) = 0.191
*S* = 1.04
2002 reflections
166 parameters
1 restraint
H atoms treated by a mixture of independent and constrained refinement
Δρ_max_ = 0.18 e Å^−3^
Δρ_min_ = −0.21 e Å^−3^

### 

Data collection: *COLLECT* (Nonius, 1998 ▶); cell refinement: *XSCANS* (Bruker, 2000 ▶); data reduction: *XSCANS*; program(s) used to solve structure: *SHELXS97* (Sheldrick, 2008 ▶); program(s) used to refine structure: *SHELXL97* (Sheldrick, 2008 ▶); molecular graphics: *Mercury* (Macrae *et al.*, 2006 ▶); software used to prepare material for publication: *SHELXL97*.

## Supplementary Material

Crystal structure: contains datablock(s) I, global. DOI: 10.1107/S1600536811052275/fj2486sup1.cif

Structure factors: contains datablock(s) I. DOI: 10.1107/S1600536811052275/fj2486Isup2.hkl

Supplementary material file. DOI: 10.1107/S1600536811052275/fj2486Isup3.cml

Additional supplementary materials:  crystallographic information; 3D view; checkCIF report

## Figures and Tables

**Table 1:** Hydrogen-bond geometry (Å, °)

*D*—H⋯*A*	*D*—H	H⋯*A*	*D*⋯*A*	*D*—H⋯*A*
O2—H⋯O1^i^	0.85 (1)	1.79 (2)	2.601 (3)	159 (4)

Symmetry code: (i) 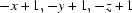.

**Fig. 1.**
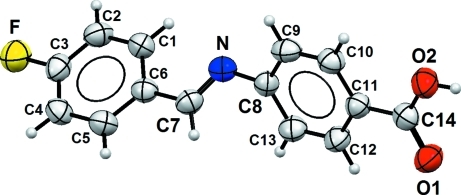
The molecular structure of (I), with atom labels and 50% probability displacement ellipsoids for non-H atoms.

**Fig. 2.**
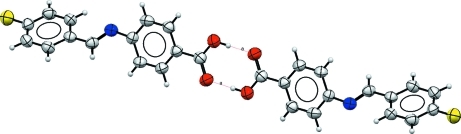
Intermolecular interaction via hydrogen bonds.

## supplementary crystallographic information

### Comment

Hugo Josef Schiff discovered the condensation a primary amine and a carbonyl compounds (Schiff 1864) afford the corresponding azomethine group. This organic compounds show an important synthetic advantages such as: high yields, simple synthetic route, short-time reactions, and easy isolation. Schiff base compounds have been of great importance in coordination chemistry (Borisova *et al.* 2007) due to the lone pair of electrons in an *sp*^2^ hybridized orbital of nitrogen atom of the azomethine group. On the other hand, it has been reported that organic compounds containing donor and acceptor groups linked through to pi-system delocalized exhibit promising nonlinear optical properties (Innocenzi & Lebeau 2005). We have been focused our attention on synthesis of *push-pull* organic molecules with no linear optical potential properties (Muñoz-Flores *et al.* 2008). In continuous with our research, we synthesized the title compound (*E*)-4-(4-fluorobenzylideneamino)benzoic acid by condensation of 4-fluorobenzaldehyde and 4-aminobenzoic acid. In the present article, the crystal structure of (I) is being reported as shown in Fig 1. The compound, (*E*)-4-(4-fluorbenzylideneamino)benzoic acid (C_14_H_10_N_2_O_2_F), displays *C*_1_ symmetry. The aromatic rings are not in the same plane, with a dihedral angle of 15.59° between mean planes. The carboxylic group represents a delocalized system with C(14)—O(1) and C(14)—O(2) bond lengths are 1.275 (4) and 1.293 (4) Å, respectively. The azomethine group are in the same plane as the monofluorated ring, probably because the short contact between the C(5)—H(5)···N(1) 2.633 (4) Å, [〈 C—H···N: 96.8°]. The intermolecular O(2)—H(2)···O(1) [2.621 (5)Å (angleO-H···O: 156.30°], hydrogen bond form a dimer with a inversion center as shown in Fig 2.

### Experimental

A solution of 4-fluorobenzaldehyde (0.5 g, 4 mmol) and 4-aminobenzoic acid (0.55 g, 4 mmol) in benzene (50 ml) was heated under reflux for 6 h, with a Dean-Stark apparatus used for the azeotropic removal of water and allowed to cool to room temperature. Removal of solvent yielded a pale yellow solid, which was recrystallized from hot benzene (10 ml). Yield: 0.74 g 76%. *M*. p. 191 °C. 1H NMR (400.13 MHz, MeOD): δ = 4.9 (bs, 1H, OH), 6.62 (d, ^3^*J* = 8.4 Hz, 2H, H-11/H-12), 6.80 (d, ^3^*J* = 8.4 Hz, 2H, H-9/H-13), 7.10 (t, ^3^*J* = 8.4 Hz, 2H, H-2/H-4), 8.05 (d, ^3^*J* = 78.4 Hz, 2H, H-1/H-5), 8.53 (s, 1H, H-7). MS (DIP 20 eV) for C_14_H_10_N_2_O_2_F (f. w: 243.24 g/mol) *m/z* (%): 243 (100) [*M*^+^], 226 (3) [*M*^+^—H_2_O], 198 (3) [*M*^+^—CO_2_], 137 (4) [*M*^+^—FC_6_H_8_], 121 [*M*^+^—C_7_H_6_O_2_].

### Refinement

All C-bonded H atoms were placed in calculated positions and refined as riding to their carrier atoms, with bond lengths fixed to 0.93 (aromatic CH). Isotropic displacement parameters for H atoms were calculated as *U*_iso_(H) = 1.5*U*_eq_(carrier atom.

### Crystal data

**Table:**

C_14_H_10_FNO_2_	*F*(000) = 504
*M**_r_* = 243.24	*D*_x_ = 1.406 Mg m^−^^3^
Monoclinic, *P*2_1_/*n*	Melting point: 464 K
Hall symbol: -P 2yn	Mo *K*α radiation, λ = 0.71073 Å
*a* = 12.2787 (5) Å	Cell parameters from 4962 reflections
*b* = 5.6264 (2) Å	θ = 2.9–27.5°
*c* = 17.2874 (8) Å	µ = 0.11 mm^−^^1^
β = 105.833 (2)°	*T* = 293 K
*V* = 1148.99 (8) Å^3^	Prism, yellow
*Z* = 4	0.23 × 0.2 × 0.15 mm

### Data collection

**Table:**

Nonius KappaCCD diffractometer	1226 reflections with *I* > 2σ(*I*)
Radiation source: fine-focus sealed tube	*R*_int_ = 0.186
graphite	θ_max_ = 25.0°, θ_min_ = 3.7°
CCD rotation images, thick slices scans	*h* = −14→14
9975 measured reflections	*k* = −6→6
2002 independent reflections	*l* = −17→20

### Refinement

**Table:**

Refinement on *F*^2^	Primary atom site location: structure-invariant direct methods
Least-squares matrix: full	Secondary atom site location: difference Fourier map
*R*[*F*^2^ > 2σ(*F*^2^)] = 0.062	Hydrogen site location: inferred from neighbouring sites
*wR*(*F*^2^) = 0.191	H atoms treated by a mixture of independent and constrained refinement
*S* = 1.04	*w* = 1/[σ^2^(*F*_o_^2^) + (0.0979*P*)^2^ + 0.0699*P*] where *P* = (*F*_o_^2^ + 2*F*_c_^2^)/3
2002 reflections	(Δ/σ)_max_ < 0.001
166 parameters	Δρ_max_ = 0.18 e Å^−^^3^
1 restraint	Δρ_min_ = −0.21 e Å^−^^3^

### Fractional atomic coordinates and isotropic or equivalent isotropic displacement parameters (Å^2^)

**Table:**

	*x*	*y*	*z*	*U*_iso_*/*U*_eq_	
C1	0.7423 (2)	0.3339 (5)	−0.03261 (18)	0.0515 (8)	
H1	0.7623	0.4696	−0.0012	0.062*	
C2	0.7740 (2)	0.3115 (5)	−0.10261 (19)	0.0565 (8)	
H2	0.8169	0.4284	−0.1185	0.068*	
C3	0.7407 (3)	0.1119 (5)	−0.14822 (19)	0.0557 (8)	
C4	0.6800 (2)	−0.0683 (5)	−0.12777 (19)	0.0567 (8)	
H4	0.6588	−0.2005	−0.1607	0.068*	
C5	0.6511 (2)	−0.0474 (5)	−0.05617 (18)	0.0528 (7)	
H5	0.6116	−0.1698	−0.0397	0.063*	
C6	0.6803 (2)	0.1547 (4)	−0.00823 (17)	0.0479 (7)	
C7	0.6428 (2)	0.1789 (5)	0.06460 (17)	0.0506 (7)	
H7	0.6186	0.0437	0.0861	0.061*	
C8	0.6104 (2)	0.3845 (4)	0.17270 (17)	0.0452 (7)	
C9	0.5490 (2)	0.5794 (4)	0.1872 (2)	0.0563 (8)	
H9	0.5247	0.6939	0.1474	0.068*	
C10	0.5237 (2)	0.6054 (5)	0.25934 (19)	0.0534 (8)	
H10	0.4824	0.7367	0.2677	0.064*	
C11	0.5597 (2)	0.4361 (4)	0.32045 (17)	0.0482 (7)	
C12	0.6204 (2)	0.2400 (5)	0.30522 (19)	0.0554 (8)	
H12	0.6446	0.1251	0.3449	0.066*	
C13	0.6449 (2)	0.2136 (5)	0.23337 (18)	0.0546 (8)	
H13	0.6849	0.0808	0.2246	0.065*	
C14	0.5353 (2)	0.4651 (5)	0.39801 (18)	0.0523 (8)	
F1	0.77081 (18)	0.0920 (3)	−0.21809 (12)	0.0827 (7)	
N1	0.64180 (18)	0.3768 (4)	0.10000 (15)	0.0530 (7)	
O1	0.56240 (18)	0.3041 (4)	0.45179 (13)	0.0679 (7)	
O2	0.48502 (19)	0.6570 (4)	0.41100 (13)	0.0675 (7)	
H	0.487 (3)	0.678 (7)	0.4597 (9)	0.101*	

### Atomic displacement parameters (Å^2^)

**Table:**

	*U*^11^	*U*^22^	*U*^33^	*U*^12^	*U*^13^	*U*^23^
C1	0.0568 (16)	0.0450 (14)	0.0523 (19)	−0.0016 (12)	0.0142 (13)	−0.0017 (12)
C2	0.0623 (17)	0.0512 (15)	0.061 (2)	0.0016 (13)	0.0245 (15)	0.0098 (14)
C3	0.0649 (18)	0.0582 (17)	0.0471 (19)	0.0168 (14)	0.0206 (14)	0.0037 (14)
C4	0.0639 (17)	0.0483 (15)	0.058 (2)	0.0042 (13)	0.0177 (15)	−0.0075 (14)
C5	0.0526 (15)	0.0457 (14)	0.062 (2)	−0.0007 (12)	0.0191 (13)	0.0006 (14)
C6	0.0455 (14)	0.0456 (14)	0.0520 (19)	0.0034 (11)	0.0122 (13)	0.0026 (12)
C7	0.0508 (15)	0.0520 (15)	0.0516 (18)	−0.0003 (12)	0.0185 (13)	0.0061 (13)
C8	0.0433 (14)	0.0493 (15)	0.0436 (17)	−0.0052 (11)	0.0131 (12)	−0.0007 (12)
C9	0.0569 (16)	0.0434 (14)	0.066 (2)	0.0042 (12)	0.0116 (14)	0.0095 (13)
C10	0.0558 (16)	0.0470 (14)	0.058 (2)	0.0056 (12)	0.0165 (14)	0.0001 (13)
C11	0.0478 (14)	0.0478 (14)	0.0477 (18)	0.0006 (11)	0.0106 (12)	0.0016 (13)
C12	0.0628 (17)	0.0532 (16)	0.0474 (18)	0.0097 (13)	0.0106 (14)	0.0066 (13)
C13	0.0551 (16)	0.0503 (15)	0.056 (2)	0.0109 (12)	0.0112 (14)	0.0044 (13)
C14	0.0467 (14)	0.0511 (15)	0.056 (2)	0.0011 (13)	0.0085 (12)	−0.0023 (14)
F1	0.1162 (16)	0.0737 (12)	0.0682 (14)	0.0116 (11)	0.0420 (12)	0.0015 (10)
N1	0.0532 (14)	0.0494 (13)	0.0564 (16)	0.0016 (10)	0.0150 (11)	0.0027 (11)
O1	0.0832 (15)	0.0652 (13)	0.0558 (15)	0.0141 (11)	0.0198 (11)	0.0101 (10)
O2	0.0804 (15)	0.0660 (13)	0.0549 (15)	0.0158 (10)	0.0165 (12)	−0.0040 (11)

### Geometric parameters (Å, °)

**Table:**

C1—C2	1.375 (4)	C8—C13	1.400 (4)
C1—C6	1.396 (4)	C8—N1	1.413 (4)
C1—H1	0.9300	C9—C10	1.372 (4)
C2—C3	1.369 (4)	C9—H9	0.9300
C2—H2	0.9300	C10—C11	1.402 (4)
C3—C4	1.360 (4)	C10—H10	0.9300
C3—F1	1.361 (4)	C11—C12	1.396 (4)
C4—C5	1.383 (4)	C11—C14	1.460 (4)
C4—H4	0.9300	C12—C13	1.363 (4)
C5—C6	1.395 (4)	C12—H12	0.9300
C5—H5	0.9300	C13—H13	0.9300
C6—C7	1.460 (4)	C14—O1	1.275 (4)
C7—N1	1.272 (3)	C14—O2	1.294 (3)
C7—H7	0.9300	O2—H	0.845 (10)
C8—C9	1.392 (4)		
			
C2—C1—C6	120.6 (3)	C9—C8—N1	118.5 (2)
C2—C1—H1	119.7	C13—C8—N1	123.0 (2)
C6—C1—H1	119.7	C10—C9—C8	121.0 (3)
C3—C2—C1	118.0 (3)	C10—C9—H9	119.5
C3—C2—H2	121.0	C8—C9—H9	119.5
C1—C2—H2	121.0	C9—C10—C11	120.7 (3)
C4—C3—F1	118.0 (3)	C9—C10—H10	119.7
C4—C3—C2	124.1 (3)	C11—C10—H10	119.7
F1—C3—C2	117.9 (3)	C12—C11—C10	118.0 (3)
C3—C4—C5	117.5 (3)	C12—C11—C14	121.0 (2)
C3—C4—H4	121.2	C10—C11—C14	121.0 (2)
C5—C4—H4	121.2	C13—C12—C11	121.3 (3)
C4—C5—C6	120.9 (3)	C13—C12—H12	119.4
C4—C5—H5	119.5	C11—C12—H12	119.4
C6—C5—H5	119.5	C12—C13—C8	120.7 (3)
C5—C6—C1	118.8 (3)	C12—C13—H13	119.6
C5—C6—C7	119.9 (2)	C8—C13—H13	119.6
C1—C6—C7	121.3 (2)	O1—C14—O2	120.6 (3)
N1—C7—C6	122.9 (2)	O1—C14—C11	120.8 (2)
N1—C7—H7	118.5	O2—C14—C11	118.7 (3)
C6—C7—H7	118.5	C7—N1—C8	119.7 (2)
C9—C8—C13	118.3 (3)	C14—O2—H	114 (3)
			
C6—C1—C2—C3	1.6 (4)	C9—C10—C11—C12	−0.8 (4)
C1—C2—C3—C4	−1.4 (4)	C9—C10—C11—C14	178.6 (2)
C1—C2—C3—F1	179.0 (2)	C10—C11—C12—C13	0.4 (4)
F1—C3—C4—C5	179.2 (2)	C14—C11—C12—C13	−179.0 (3)
C2—C3—C4—C5	−0.3 (4)	C11—C12—C13—C8	0.5 (4)
C3—C4—C5—C6	1.9 (4)	C9—C8—C13—C12	−1.0 (4)
C4—C5—C6—C1	−1.8 (4)	N1—C8—C13—C12	174.3 (2)
C4—C5—C6—C7	176.2 (2)	C12—C11—C14—O1	−4.7 (4)
C2—C1—C6—C5	0.0 (4)	C10—C11—C14—O1	175.9 (2)
C2—C1—C6—C7	−178.0 (2)	C12—C11—C14—O2	175.7 (2)
C5—C6—C7—N1	−162.4 (3)	C10—C11—C14—O2	−3.7 (4)
C1—C6—C7—N1	15.6 (4)	C6—C7—N1—C8	−176.5 (2)
C13—C8—C9—C10	0.6 (4)	C9—C8—N1—C7	−143.9 (3)
N1—C8—C9—C10	−174.9 (2)	C13—C8—N1—C7	40.8 (4)
C8—C9—C10—C11	0.3 (4)		

### Hydrogen-bond geometry (Å, °)

**Table:**

*D*—H···*A*	*D*—H	H···*A*	*D*···*A*	*D*—H···*A*
O2—H···O1^i^	0.85 (1)	1.79 (2)	2.601 (3)	159 (4)

Symmetry codes: (i) −*x*+1, −*y*+1, −*z*+1.
